# A Self-Powered Hybrid SSHI Circuit with a Wide Operation Range for Piezoelectric Energy Harvesting

**DOI:** 10.3390/s21020615

**Published:** 2021-01-17

**Authors:** Liao Wu, Peidong Zhu, Minghua Xie

**Affiliations:** 1 School of Electronic Information and Electrical Engineering, Changsha University, Changsha 410022, China; zpd136@sina.com (P.Z.); xieminghua@126.com (M.X.); 2 School of Automatic, Northwestern Polytechnical University, Xi’an 710072, China

**Keywords:** wide operation range, piezoelectric energy harvesting, SSHI, vibration energy harvesting

## Abstract

This paper presents a piezoelectric (PE) energy harvesting circuit, which integrates a Synchronized Switch Harvesting on Inductor (SSHI) circuit and a diode bridge rectifier. A typical SSHI circuit cannot transfer the power from a PE cantilever into the load when the rectified voltage is higher than a certain voltage. The proposed circuit addresses this problem. It uses the two resonant loops for flipping the capacitor voltage and energy transfer in each half cycle. One resonant loop is typically used for the parallel SSHI scheme, and the other for the series SSHI scheme. The hybrid SSHI circuit using the two resonant loops enables the proposed circuit’s output voltage to no longer be limited. The circuit is self-powered and has the capability of starting without the help of an external battery. Eleven simple discrete components prototyped the circuit. The experimental results show that, compared with the full-bridge (FB) circuit, the amount of power harvested from a PE cantilever and the Voltage Range of Interest (VRI) of the proposed circuit is increased by 2.9 times and by 4.4 times, respectively. A power conversion efficiency of 83.2% is achieved.

## 1. Introduction

Piezoelectric (PE) energy harvesting has attracted immense interest due to its potential for applications such as the Internet of Things (IoT) and implant devices. It is very suitable for small-scale energy harvesting due to the high power density and easy scalability of the PE transducer. The general purpose of the PE energy harvesting circuit requires: (1) rectification, (2) maximum power tracking, and (3) output voltage regulation [1]. Many researchers have focused on the first two points, rectification and maximum power tracking, due to a PE transducer’s source impedance characteristics. Various PE energy harvesting circuits to improve efficiency have been reviewed [2]. The harvesting circuit’s objective is to achieve efficient energy extraction from the PE transducer while minimizing the power dissipation of the circuit. Therefore, maintaining efficient PE energy harvesting over the PE transducer’s wide operating range is still a challenge for circuit design.

**Figure 1:**
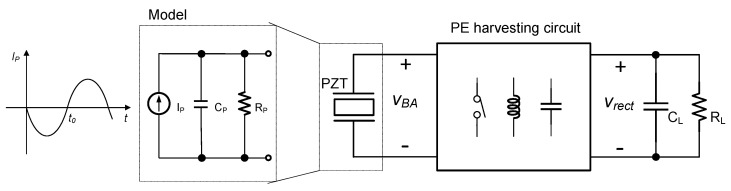
Piezoelectric (PE) energy harvesting system.

Figure 1 shows the PE energy harvesting system, which consists of the PE transducer, circuit, and a load. The PE energy harvesting circuit converts AC voltage *V_BA_* from the PE transducer into a DC output voltage *V_rect_*. The PE transducer has high output impedance with a large capacitive term. Due to this fact, various sophisticated rectifiers have been reported in the literature to improve power extraction [3,4,5,6,7,8,9,10,11,12,13,14,15,16,17,18,19,20,21,22,23,24,25,26,27,28]. One group of the PE energy harvesting circuits adopts a straightforward rectification scheme. The scheme adopts a full-bridge (FB) rectifier, which consists of four diodes and a relatively large filter capacitor, to obtain a DC voltage from the PE transducer AC voltage. The shortcoming of this circuit is the charge wasted during the rectification. Consider when the PE transducer current *i_P_* changes its polarity, say *t* = *t_0_*, from negative to positive. As the internal capacitor C_P_ of the PE transducer is previously charged negatively to store negative charge with negative voltage *V_BA_*, the positive charge generated by the PE transducer will reversely discharge the C_P_, and so the amount of charge is wasted. The output power of the circuit is dependent on the rectified voltage *V_rect_* [29].

Another group of PE energy harvesting circuits includes the synchronous switch technique for the rectification scheme. The adoption of the synchronous switch technique can enhance the electrostatic force or damping force of the PE transducer and significantly improve the power extraction efficiency. There are three typical schemes. The Synchronized Switch Shorting (SSS) scheme instantly shorts the output voltage of the PE transducer at *t_0_*, discarding the negative charge to zero voltage *V_BA_*, thereby saving reverse charge [3,4]. The SSS does not require an inductor, but the short operation wastes the previously stored charge on the capacitor C_P_, resulting in insufficient efficiency. In contrast, Synchronized Switch Harvesting on Inductor (SSHI) [6,7] and Synchronous Electric Charge Extraction (SECE) [8] harvest the charge that would have been wasted by the short operation. The SSHI and SECE schemes can harvest more energy than the SSS scheme. Compared to FB, the SSHI or SECE circuit’s output power can be increased by four times or even higher. A variety of circuits reported in the literature to improve the SSHI or SECE scheme explore the potential of efficiency improvement by using the resonant circuits formed by the external inductor and internal capacitor of the PE transducer [5,6,7,8,9,10,11,12,13,14,15,16,17,18,19,20,21,22,23,24,25,26,27].

The SSHI scheme uses a resonant loop to change the polarity of the capacitor charge at *t_0_*, which flips the capacitor voltage *V_BA_* instantly. This way, the PE transducer current charges the internal capacitor continuously instead of discharges first. A variety of improved SSHI schemes have been reported in the literature [8,9,10,11,12,13,14,15,16]. The SSHI circuit and DC-DC converter in [8] share the inductor to reduce the size. A trickle charger with the SSHI circuit charges the energy storage device [9]. The use of the SSHI scheme harvests shock vibration energy [10]. The insertion of a diode in the resonant loop for the SSHI scheme was adopted [11,12]. Re-share components to further simplify the controller were adopted in [13,14] for self-powered schemes. A triple bias-flip SSHI circuit was proposed in [15] to reduce the energy loss during the capacitor voltage flipping. The circuit was fully integrated by using on-chip capacitors instead of an external inductor to flip the capacitor voltage [16]. Unlike the SSHI scheme, the typical SECE scheme first transfers the capacitor energy to the external inductor and then to the load. Therefore, the PE transducer is open most of the time, and the power extraction for the SECE scheme is independent of the load. Based on a typical SECE scheme, a variety of improved circuits have been reported [17,18,19,20,21,22]. The SECE circuit operates over a wide operation range by fine controlling the pulse timing [17]. The SECE circuit was designed for shocked vibration energy harvesting [18], self-powered [19], and energy loss reduction [20,21]. Finally, using the transformer instead of a diode bridge [22] reduces the circuit’s power dissipation.

Theoretically, the efficiency of the SECE circuit is independent of the load. The circuit uses two resonant loops to harvest the energy per half cycle. Compared to SECE, except for those low-coupling PE transducers, SSHI is suitable for almost any type of PE transducer, which can harvest more energy. To increase the efficiency of the PE energy harvesting over a wide operation range of the PE transducer, the circuits in [23,24,25] integrated SSHI and Maximum Power Point Tracking (MPPT). The circuits in [3,25] realized a self-powered MPPT. However, it requires an additional controller, such as using the “VDD_MUX” block [3] and adopting the three different operation modes [25]. The increase in the circuit blocks would increase the circuit‘s complexity, and hence higher power dissipation. Additionally, the power dissipation for the MPPT operation increases as the source and load change rapidly. In contrast, other approaches to expand the operating range were adopted in [27,28]. Multiple resonant circuits to extract power were used in [27], or the circuit topology was reconfigured in the operation according to the PE voltage [28]. However, the control circuit for switch timing was not shown in [27], and the SSHI scheme did not use the PE interface in [28].

This paper presents a hybrid SSHI circuit which is integrated with a diode bridge rectifier. The rectification scheme implemented by the proposed circuit significantly extends the operating range of the rectified voltage for efficient PE energy harvesting. The key advantage of the proposed circuit is simple and easy to implement. The circuit is self-powered without the help of an external power supply. This paper is organized as follows. Section 2 describes the basic operation of the SSHI circuits and analyzes their maximum allowable rectified voltage. Section 3 presents the proposed rectifier scheme followed by the operation, modeling analysis, extracted power analysis, and operation range of the circuit. Section 4 presents the proposed circuit and describes its operation. Section 5 shows the measurement results of the proposed circuit and compares its performance with other recent circuits. Section 6 draws the conclusion.

## 2. Preliminaries

### 2.1. Review of Relevant SSHI Circuits

**Figure 2:**
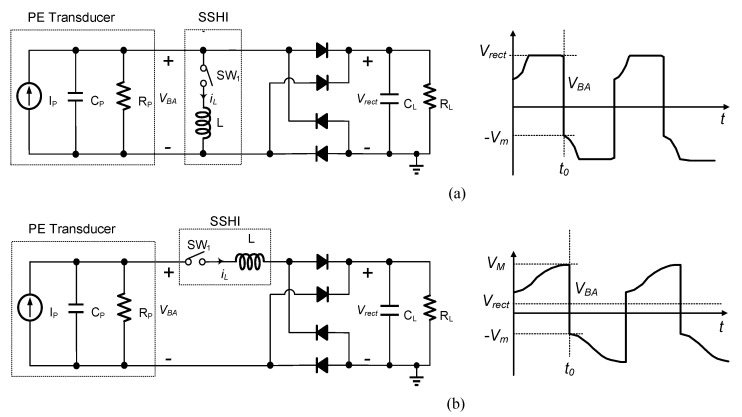
Typical (**a**) P-SSHI circuit and (**b**) S-SSHI circuit.

Figure 2 shows a typical Parallel-SSHI (P-SSHI) circuit [5] and a Series-SSHI (S-SSHI) circuit [6]. The PE transducer is modeled as a current source i_P_ in parallel with a capacitor C_P_, and a resistor R_P_, where $i_{P}=I_{P}sin\left(2\pi f_{P}t\right)$, and $f_{P}$ is the excitation frequency of the PE transducer. This simple model is suitable when the PE transducer is weakly coupled, and it is used for PE circuits’ performance analysis in this paper. Both typical P-SSHI and S-SSHI circuits incorporate a switch (SW_1_ in Figure 2) in series with an external inductor L as shown in Figure 2.

The operation of typical SSHI circuits is illustrated in Figure 2 with associated waveforms. Unlike the P-SSHI circuit, the SSHI circuit adds the load C_L_ and R_L_ in the resonant loop. Due to its series connection, the PE transducer is open most of the time, and the energy from the PE transducer is transferred into load only when the capacitor voltage is flipped. In contrast, the P-SSHI circuit transfers the energy to the load only when the rectified voltage clamps the PE voltage. The SSHI circuit harvests the charge that would have been wasted per cycle to obtain more power than the FB circuit. Unlike the SECE, the SSHI circuits use one resonant loop instead of two resonant loops to harvest the charge that would have been wasted during the rectification, which limits the rectified voltage’s range. 

The SSHI circuit requires an associated controller for switch timing. Ramadass et al. [9] used a comparator with an external reference voltage and an adjustable RC delay circuit to control the synchronous switch. Wu et al. [14] integrated the SSHI circuit with the active rectifier with the optimal flipping time. Eltamaly et al. [15] proposed a discrete self-powered switch to realize the SSHI operation. Fang et al. [24] proposed an S-SSHI circuit with the Fractional Normal-Operation Voltage (FNOV) scheme. Wu and Ha [25] adopted the MPPT circuit to extend the operating range of the SSHI circuit. The adoption of the MPPT scheme enables the SSHI circuit to operate over a wide operation range. However, the MPPT circuit requires an additional controller and hence more power dissipation. 

This paper adopts a self-powered switch proposed in [14], but with a different rectification scheme and a circuit. The proposed scheme significantly extends the operating range of the rectifier voltage for harvesting energy efficiently. Unlike the typical SSHI circuit, two resonant loops rather than one loop are used for the voltage flipping. One resonant loop is typically used for the P-SSHI scheme and for the power extraction, and the other is used for the S-SSHI scheme and for delivering the energy to the load and back to the PE transducer. This scheme is feasible as the two resonant loops switch at the inductor’s current peak, forming a boost topology.

### 2.2. Effect of the Rectifier Voltage on the Extracted Power

The SSHI circuit forms a resonant circuit to flip the internal capacitor’s voltage, and its output power is dependent on the rectified voltage *V_rect_*. If the rectified voltage is too high, the load may not obtain the power from the PE transducer. The effect of the rectified voltage on the output power of the SSHI circuit is analyzed as follows.

If the PE transducer being excited opened, its open-circuit voltage could be expressed as $V_{oc}=\frac{I_{P}}{2\pi f_{P}C_{P}}$. Let us define the flip voltage ratio *η_F_* as the ratio of the amount of the capacitor voltage change to the maximum possible voltage change for a typical P-SSHI and an S-SSHI circuit shown in Figure 2. Referring to the waveform in Figure 2, with an ideal FB rectifier, the $\eta_{F}$ of the two circuits is:

(1) $$\eta_{F}=\frac{V_{rect}+V_{m}}{2V_{rect}}\;\mathrm{for}\;P-\mathrm{SSHI},\;\eta_{F}=\frac{V_{M}+V_{m}}{2\left(V_{M}-V_{rect}\right)}\;\mathrm{for}\;S-\mathrm{SSHI}$$

The output power of the P-SSHI and the S-SSHI circuit can be obtained as:

$$P_{out,P-SSHI}=4f_{P}C_{P}\left[V_{oc}-\left(1-\eta_{F}\right)V_{rect}\right]\cdot V_{rect}$$

(2) $$P_{out,S-SSHI}=4f_{P}C_{P}\frac{\eta_{F}}{1-\eta_{F}}\left(V_{oc}-V_{rect}\right)\cdot V_{rect}$$

According to Equation (2), the optimal rectified voltage *V_rect,opt_* for the maximum power is obtained as:

(3) $$V_{rect,opt,P-SSHI}=\frac{V_{oc}}{2\left(1-\eta_{F}\right)},\;V_{rect,opt,S-SSHI}=\frac{V_{oc}}{2}$$

**Figure 3:**
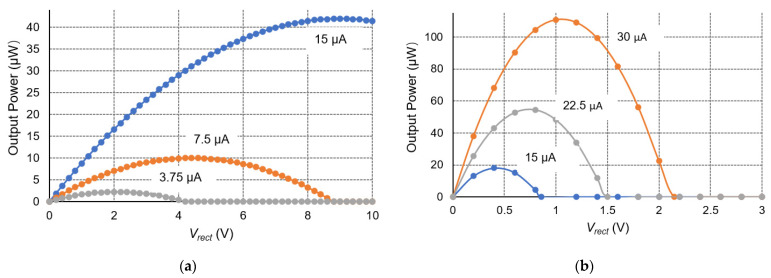
Output power versus *V_rect_* for a typical (**a**) P-SSHI circuit and (**b**) S-SSHI circuit.

Since the output power *P_out_* is a quadratic function of the rectified voltage *V_rect_* within the operation range, the maximum allowable rectified voltage *V_rect,max_* of the P-SSHI and S-SSHI circuits is twice the optimal rectified voltage *V_rect,opt_*. If the rectified voltage exceeds *V_rect,max_*, the load cannot obtain any power from the PE transducer, which is the common shortcoming for both SSHI circuits. Figure 3 shows the relationship between output power and rectified voltage for a typical P-SSHI circuit and a typical S-SSHI circuit shown in Figure 2, at the excitation frequency of 100 Hz with different excitation amplitude.

With maintaining a high PE power extraction efficiency, the rectified voltage is required to be adjusted within a specific range. Let us define the Voltage Range of Interest (VRI) as:

(4) $$VRI=\frac{V_{h}-V_{l}}{V_{oc}}$$

where *V_h_* and *V_l_* represent the upper and lower threshold of the rectified voltage, respectively, and their corresponding output power of the circuit is *1/m* times the maximum available power, which is expressed as $P_{out}\left(V_{h}\right)=P_{out}\left(V_{l}\right)=\frac{P_{out,max}}{m}$.

According to Equation (2), the VRI for the two typical SSHI circuits can be obtained as:

(5) $$VRI_{P-SSHI}=\frac{\sqrt{1-\frac{1}{m}}}{1-\eta_{F}},VRI_{S-SSHI}=\sqrt{1-\frac{1}{m}}$$

The VRI for the P-SSHI circuit is related to the voltage flipping ratio $\eta_{F}$. In contrast, the S-SSHI circuit is independent of the $\eta_{F}$. As $\eta_{F}$ increases, the VRI of the P-SSHI circuit increases, but not for the S-SSHI circuit. 

As only one resonant loop is used for voltage flipping for typical SSHI circuits, the VRI is limited. The proposed circuit in this paper uses two resonant loops for the flipping of the capacitor voltage. One loop is used for extracting power, and the other loop is used for delivering the energy to the load and also back to the PE transducer. The proposed circuit operated in two different modes according to the PE voltage. Even with the relatively high rectified voltage, the circuit can also deliver the energy to the load due to the two resonant loops’ boost operation. 

## 3. Proposed Rectifier Scheme

### 3.1. Block Diagram

**Figure 4:**
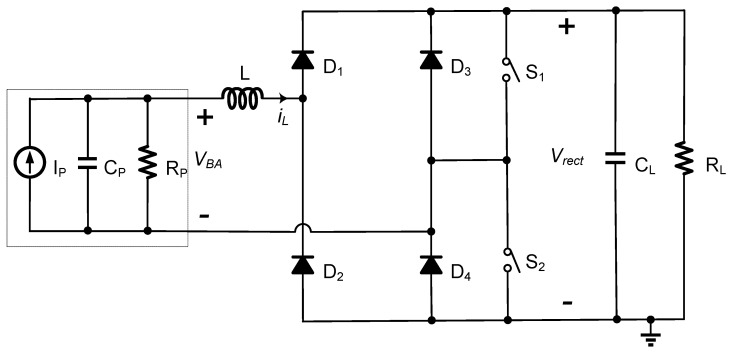
Proposed hybrid Synchronized Switch Harvesting on Inductor (SSHI) circuit integrated with a diode bridge rectifier.

Figure 4 shows the block diagram of the proposed hybrid SSHI circuit, which is integrated with a diode bridge rectifier. The proposed circuit consists of an FB rectifier with four diodes D_1_–D_4_, two switches S_1_–S_2_, and one inductor L. The rectified voltage *V_rect_* is constant due to the relatively large capacitor C_L_. To simplify the analysis and illustration, assume that the diodes and switches are ideal unless otherwise stated.

### 3.2. Operation

**Figure 5:**
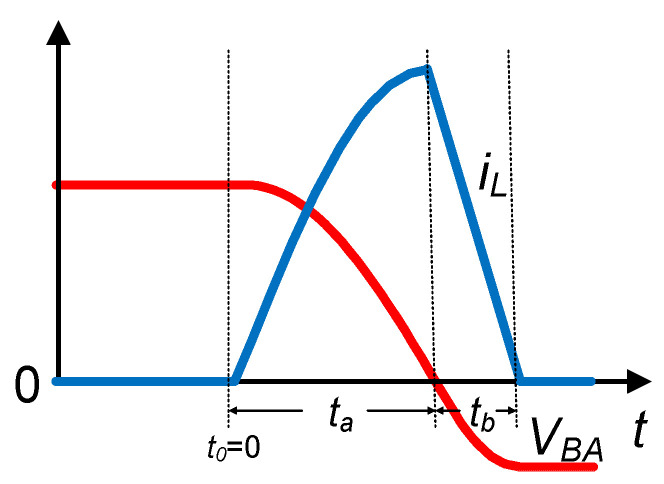
Waveform of the voltage *V_BA_* and inductor current *i_L._*

Figure 5 shows the waveforms of voltage *V_BA_* and inductor current *i_L_* when the proposed SSHI circuit operates for voltage flipping. Refer to Figure 4 for circuit operation. When the current *i_P_* crosses the zero point from positive to negative, the switch S_1_ is turned on, forming one resonant loop through the devices of C_P_-L-D_1_-S_1_-C_P_. The energy in the PE transducer is transferred to the inductor over the time interval of *t_a_*. When the inductor current *i_L_* reaches the peak value, the S_1_ is turned off, and the other resonant loop is formed through the devices of C_P_-L-D_1_-C_L_ and R_L_-D_4_-C_P_. The inductor’s energy is transferred to the load during the time interval of *t_b_* and transferred back to the capacitor C_P_, with a flipping voltage on the C_P_. When the *i_P_* crosses the zero from negative to positive, the symmetrical circuit topology follows the same operating principle but uses different resonance circuits. The circuit operates in two different modes, named the P-SSHI mode and the S-SSHI mode, according to whether the PE voltage is clamped by rectified voltage *V_rect_* in each half cycle. Note that the operation of the proposed circuit in the P-SSHI or S-SSHI mode is different from that of the typical P-SSHI or S-SSHI circuits.

**Figure 6:**
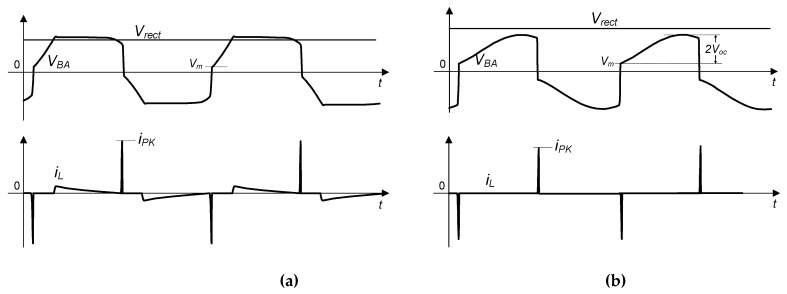
Waveform of the PE voltage *V_BA_* and inductor current *i_L_* (**a**) in the P-SSHI mode, and (**b**) in the S-SSHI mode.

Figure 6 shows the waveforms of the voltage *V_BA_* and current *i_L_* of the circuit in two different modes. As shown in Figure 6a, the proposed circuit operates in the P-SSHI mode. In this mode, the energy is transferred to the load either in the period when the FB rectifier works or when the resonant circuits run. However, if the proposed circuit is in the S-SSHI mode, the energy is transferred only in the period when the resonant circuits run, as the PE voltage cannot attain the rectified voltage of *V_rect_*, as shown in Figure 6b. The circuit’s two operating modes depend on the open circuit voltage *V_oc_*, the voltage flipping process, and the rectified voltage *V_rect_*. In Section 3.3, two resonant loops used during the voltage flipping are modeled to analyze the circuit’s operation mechanism.

### 3.3. Modelling Analysis

The proposed circuit completes the voltage flipping using the two resonant loops, whatever the operation modes. Each resonant circuit is modeled using ideal electronic components except the forward voltage drop of *V_D_* for analysis. Suppose the PE transducer current *i_P_* is zero when it begins the voltage flipping, and the R_P_ is so large that it can be ignored.

**Figure 7:**
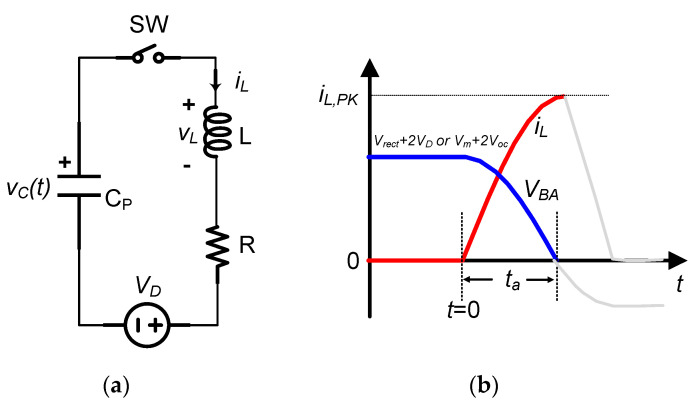
(**a**) Circuit model of the first resonant loop, and (**b**) its waveform.

- First resonant loop:

Figure 7 shows the operation of the first resonant loop to extract power from the PE transducer. Here, the switch SW represents the switch S_1_ or S_2_ in Figure 4, and *V_D_* models the forward voltage of the one diode inserted in the resonant loop. When the switch SW in Figure 7 is ON, using Kirchhoff’s Voltage Law, KVL equation of the circuit model in Figure 7a is:

(6) $$v_{C}=LC_{P}\frac{d^{2}v_{C}}{dt^{2}}+RC_{P}\frac{dv_{C}}{dt}+V_{D}$$

As $i_{L}\left(0\right)=0$, the capacitor voltage *v_C_*, i.e., *V_BA_*, the inductor voltage *v_L_*, and the inductor current *i_L_* are readily obtained as:

$$v_{C}\left(t\right)=V_{D}+\left(v_{C}\left(0\right)-V_{D}\right)\frac{\omega_{o}}{\omega }e^{-\delta t}\sin\left(\omega t+\beta \right)$$

$$i_{L}\left(t\right)=C_{P}\frac{dv_{C}}{dt}=\frac{v_{C}\left(0\right)-V_{D}}{\omega L}e^{-\delta t}\sin\left(\omega t\right)$$

(7) $$v_{L}\left(t\right)=-\frac{\omega_{0}}{\omega }\left(v_{C}\left(0\right)-V_{D}\right)e^{-\delta t}\sin\left(\omega t-\beta \right)$$

where $\beta =\arcsin\left(\frac{\omega }{\omega_{o}}\right)$, $\delta =\frac{R}{2L}$, $\omega =\sqrt{\omega_{o}^{2}-\delta^{2}}$, and $\omega_{o}=\frac{1}{\sqrt{{\mathrm{LC}}_{P}}}$. R is the total parasitic resistance along the resonant loop. Note that the $v_{C}\left(0\right)$ is the initial condition of the capacitor C_P_, which is dependent on the operation mode of the circuit.

At $t_{a}=\frac{\beta }{\omega }$, the inductor voltage $v_{L}\left(t_{a}\right)=0$, and so we obtain the capacitor voltage $v_{C}\left(t_{a}\right)=V_{C0}$ and the peak inductor current $i_{L,PK}$

(8) $$i_{L,PK}=i_{L}\left(t_{a}\right)=\frac{v_{C}\left(0\right)-V_{D}}{\omega_{o}L}e^{-\delta \cdot t_{a}}\approx \frac{v_{C}\left(0\right)-V_{D}}{\omega_{o}L}$$

**Figure 8:**
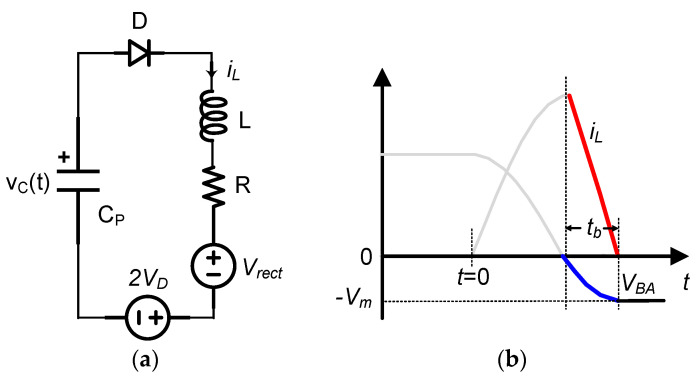
(**a**) Model of the second resonant loop, and (**b**) its waveform.

- Second resonant loop:

Figure 8 shows the operation of the second resonant loop switched from the previous first resonant circuit. Similarly, the KVL equation of the circuit model in Figure 8a is:

(9) $$v_{C}=LC_{P}\frac{d^{2}v_{C}}{dt^{2}}+RC_{P}\frac{dv_{C}}{dt}+V_{rect}+2V_{D}$$

Here, the forward voltage drop of the two diodes is considered. Since the initial capacitor voltage and inductor current are *V_C0_* and *i_L,PK_*, respectively, the voltage on the capacitor and the current through the inductor can be obtained as:

$$v_{C}\left(t\right)=V_{rect}+2V_{D}+A\cdot e^{-\delta t}\sin\left(\omega t+\varphi \right)$$

(10) $$i_{L}\left(t\right)=C_{P}\cdot A\cdot \omega_{o}e^{-\delta t}\sin\left(\omega t+\varphi -\beta \right)$$

where, $A=\sqrt{{\left(\frac{C_{P}\delta \left(V_{C0}-V_{rect}-2V_{D}\right)-iL,PK}{\omega C_{P}}\right)}^{2}+{\left(V_{C0}-V_{rect}-2V_{D}\right)}^{2}}$, and $\varphi =\pi -\arcsin\left(\frac{V_{C0}-V_{rect}-2V_{D}}{A}\right)$.

From Equation (10), when $t_{b}=\frac{\pi +\beta -\varphi }{\omega }$, the inductor current $i_{L}\left(t_{b}\right)=0$. Finally, the flipping voltage *V_m_* can be obtained as:

(11) $$V_{m}=-v_{C}\left(t_{b}\right)=A\frac{\omega }{\omega_{o}}e^{-\delta \cdot t_{b}}-V_{rect}-2V_{D}$$

- Operation mode:

The proposed circuit’s operation mode depends on whether the PE voltage is clamped by the rectified voltage *V_rect_*. If the circuit works in the P-SSHI mode, the PE voltage is clamped by *V_rect_* per half cycle, and the initial capacitor voltage $v_{C}\left(0\right)=V_{rect}+2V_{D}$. If the circuit works in the S-SSHI mode, the PE voltage cannot attain the *V_rect_*, and so the initial capacitor voltage $v_{C}\left(0\right)=V_{m}+2V_{oc}$. Therefore, the critical condition of the two modes is:

(12) $$V_{rect}+2V_{D}=V_{m}+2V_{oc}$$

Considering that the parasitic resistance R along the resonant loop is very small, say $V_{C0}\approx 0$, and $i_{L,PK}\approx \frac{V_{m}+2V_{\mathrm{oc}}}{\omega_{o}L}$. According to Equation (11), the voltage *V_m_* can be obtained as:

(13) $$V_{m}\approx \frac{{\left(2V_{oc}-V_{D}\right)}^{2}}{2\left(V_{rect}+3V_{D}-2V_{oc}\right)}$$

The critical rectified voltage $V_{rect,cri}$ between the two operation modes is approximately $\left(2+\sqrt{2}\right)V_{oc}-2V_{D}$.

Therefore, when the rectified voltage is lower than the critical rectified voltage, the PE voltage is clamped by the rectified voltage per cycle. In the P-SSHI mode, the proposed circuit operation is similar to the P-SSHI circuit, but the energy delivery to the load occurs when the FB rectifier works and the capacitor voltage flips. In contrast, in the S-SSHI mode, the energy delivery to the load occurs when the resonant circuits run only, in which the PE voltage is boosted even if the rectified voltage is quite high.

### 3.4. Extracted Power

The extracted power of the proposed circuit is analyzed in the P-SSHI or the S-SSHI mode as follows.

- P-SSHI mode:

In the P-SSHI mode, the extracted power of the two parts needs to be taken into account. One part is the power obtained by the load when the FB rectifier works. The other part is when the resonant circuits run. Let us calculate the power obtained by the load when the FB works first.

The total charge $Q_{total}$ generated by the PE transducer per cycle is $4C_{P}V_{oc}$. When the FB rectifier blocks, the charge lost $Q_{loss}$ due to charging and discharging the internal capacitor per cycle is $2C_{P}\left(V_{rect}+2V_{D}-V_{m}\right)$. So, the available charge per cycle by the load can be expressed as:

(14) $$Q_{rect}=Q_{total}-Q_{loss}=2C_{P}\left(2V_{oc}-V_{rect}-2V_{D}+V_{m}\right)$$

Thus, when the FB rectifier works, the output power of the proposed circuit in the P-SSHI mode is:

(15) $$P_{out,P-SSHI,FB}=f_{P}Q_{rect}V_{rect}=2f_{P}C_{P}\left(2V_{oc}-V_{rect}-2V_{D}+V_{m}\right)V_{rect}$$

Since the energy is also transferred to the load when the resonant circuits run, its output power is obtained as:

(16) $$P_{out,P-SSHI,flipped}=2f_{P}Q_{m}V_{rect}=2f_{P}C_{P}\left(V_{m}+V_{C0}\right)V_{rect}$$

where $Q_{m}\;$ is the total amount of charge during the interval of operation of the two resonant circuits. Since $V_{C0}\approx 0$, the total output power of the circuit in the P-SSHI mode is:

(17) $$P_{out,P-SSHI}=P_{out,P-SSHI,FB}+P_{out,P-SSHI,flipped}=2f_{P}C_{P}\left[\left(2\left(V_{oc}+V_{m}-V_{D}\right)-V_{rect}\right)V_{rect}\right]$$

- S-SSHI mode:

In the S-SSHI mode, the energy is obtained by the load only when the resonant circuits run. According to Equation (13), the output power is obtained as:

(18) $$P_{out,S-SSHI}=2f_{P}Q_{m}V_{rect}=f_{P}C_{P}V_{rect}\frac{{\left(2V_{oc}-V_{D}\right)}^{2}}{V_{rect}+3V_{D}-2V_{oc}}$$

We built the circuit shown in Figure 4 and used the LTspice software to verify the theoretical analysis. Ideal switches and diodes with *V_D_* = 0.2 V were used for the simulation. The simulation parameters are shown in Table 1.

**Figure 9:**
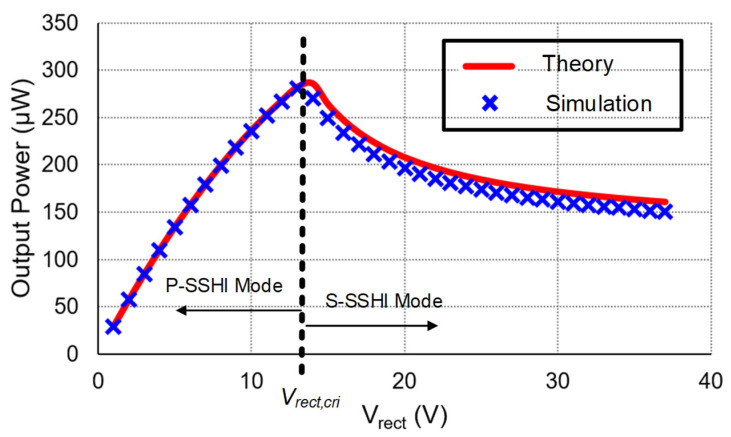
Comparison of theoretical results with simulation results.

Figure 9 compares the theoretical and simulation results. The critical rectified voltage *V_rect,cri_* distinguished the two different operation modes. As the approximation of the *V_m_* in Equation (13), it has a slight deviation between the theoretical and the simulation results in the S-SSHI mode. The optimal rectified voltage is near *V_rect,cri_*, in which the circuit can harvest maximum power harvested from the PE transducer. Unlike the typical SSHI circuit, the proposed circuit has a wide operation range of the rectified voltage in the S-SSHI mode to efficiently extract power from the PE transducer. The ideal SECE circuit’s output power is independent of the load, and its output power $P_{out,SECE}=\frac{I_{P}^{2}}{\pi^{2}f_{P}C_{P}}$ is four times the output power of the FB, which is 133 μW using the parameters in Table 1. In this case, the proposed circuit’s output power is higher than that of the SECE when the rectified voltage $V_{rect}$ is higher than 5 V. Theoretically, compared to Equations (17) and (18), after calculation, if $V_{rect}$> 1.1$V_{oc}$, the proposed circuit’s output power is larger than that of the SECE, in which the proposed circuit operates either in the P-SSHI mode or in the S-SSHI mode. However, in practice, the loss due to electronic devices of non-idealities will cause their results to deviate from the theoretical value, which will be further explained in Section 5.

### 3.5. Operation Range

**Figure 10:**
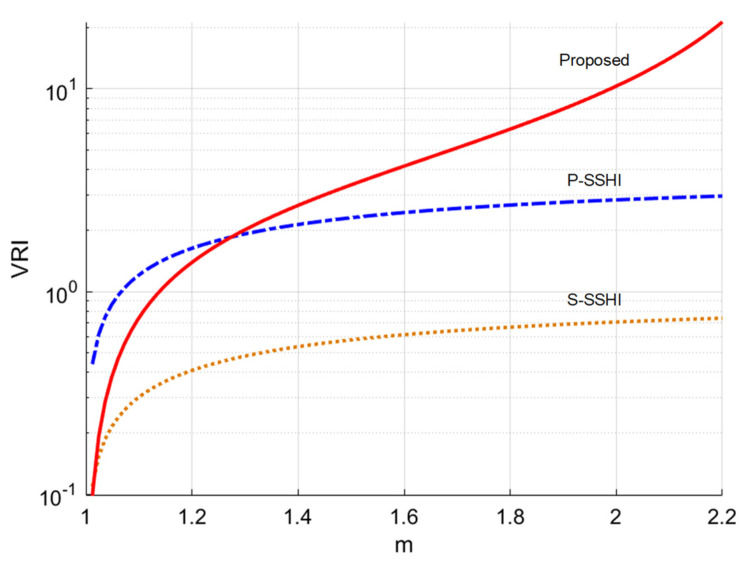
VRI versus *m.*

Table 2 shows the VRI expression for the typical P-SSHI circuit, the typical S-SSHI circuit, and the proposed circuit. Figure 10 shows the VRI versus *m* for three different circuits. Here, the *η_F_* of P-SSHI is set to 0.75. Note that *V_D_* = 0 in the analysis. As *m* > 1.3, the VRI of the proposed circuit is higher than that of the other two circuits, mainly due to the fact that the circuit can boost the rectified voltage. The VRI of the proposed circuit is smaller than the VRI of the P-SSHI circuit when *m* < 1.3 because the flip factor *η_F_* of the proposed circuit operating in the P-SSHI mode is limited 0.707.

## 4. Circuit Implementation

**Figure 11:**
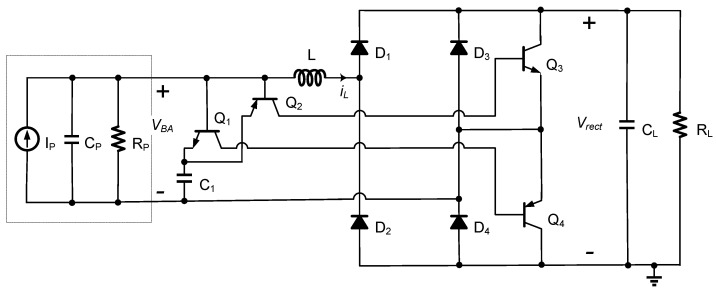
Proposed hybrid SSHI circuit integrated with a diode bridge rectifier.

Figure 11 shows the implementation for the proposed circuit, which includes a self-powered switch, an FB rectifier, and an inductor. The self-powered switch consists of two PNP transistors of Q_2_ and Q_4_, two NPN transistors of Q_1_ and Q_3_, and a capacitor C_1_. The key advantage of this circuit is simplicity. The transistors Q_1_ and Q_2_ with the capacitor C_1_ are used to detect the voltage peak of the PE voltage *V_BA_* to enable the transistors Q_3_ and Q_4_. The transistor Q_3_ or Q_4_ can be turned off itself once the inductor current approaches the peak. The detail of the operation principle is briefly described as follows.

Before the PE transducer current *i_P_* crosses the zero point from positive to negative, the NPN transistor’s base-emitter of Q_1_ is turned on to make the capacitors C_1_ and C_P_ connect in parallel. As the current *i_P_* crosses the zero point to discharge the capacitor C_P_, and the voltage *V_BA_* drops, since the voltage of the capacitor C_1_ remains unchanged, the switch Q_1_ is turned off and Q_2_ is turned on. This results in Q_3_ being turned on. It transfers the energy previously stored in the capacitor C_P_ to the inductor L using the first resonant loop formed by internal capacitor C_P_ and the inductor L, and so the inductor current *i_L_* increases. When the *i_L_* approaches its peak, the voltages across the inductor L and the capacitor C_P_ are both approximately zero, forcing Q_3_ to be turned off in the resonant loop. Once Q_3_ is off, the second resonant loop is formed by the source, the inductor L, and the load. Its operation is already stated in Section 3.2. The rectified voltage could be higher than the PE voltage without being clamped due to the use of the second resonant loop. Due to the symmetrical circuit topology, the proposed circuit follows the same operating principle but uses different resonant circuits when the current *i_P_* crosses the zero point from negative to positive.

## 5. Experiment Results

A printed circuit board (PCB) was prototyped to verify the operation and performance of the proposed circuit. The prototype circuit board was populated by only eleven components. Four Schottky diodes of SS14 with the maximum forward voltage drop of 0.6 V at room temperature were used to build the FB rectifier. The two PNP transistors of 2N3906s with *V_CE(SAT)_* = 0.25 V, *V_BE(SAT)_* = −0.65 V, and two NPN transistors of model 2N3904s with *V_CE(SAT)_* = 0.2 V, *V_BE(SAT)_* = 0.65 V, were used for Q_1_−Q_4_. The capacitor C_d_ value is selected as 1 nF, the inductor L = 1.5 mH and C_L_ = 10 μF in this paper, unless otherwise stated.

**Figure 12:**
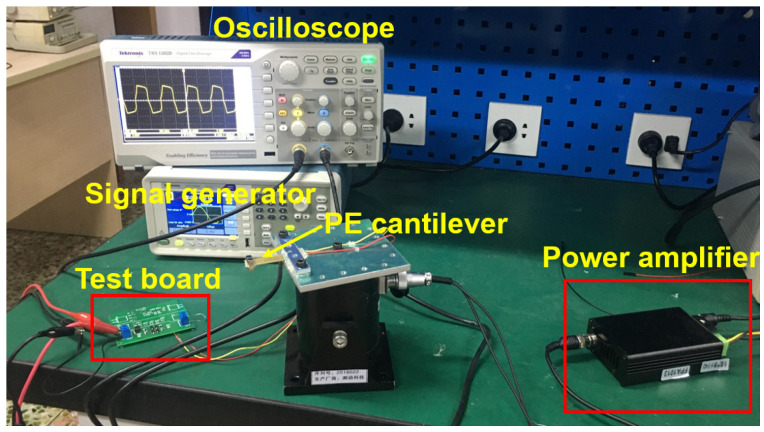
Experimental setup.

A PE cantilever was used for experimental testing. A tip mass of 1.5 g was placed at the end of the PE cantilever, and the measured internal capacitor C_P_ = 22 nF. The PE cantilever was put on a thick aluminum plate, which was mounted on the vibration shaker, as shown in Figure 12.

**Figure 13:**
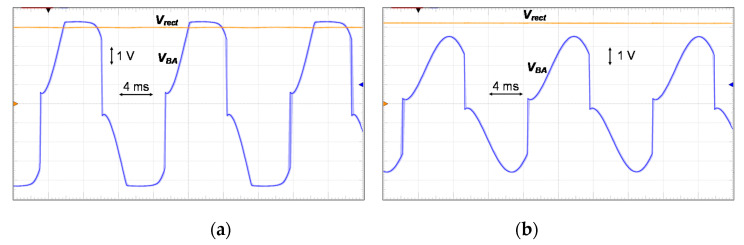
Measured waveform of the PE voltage *V_BA_* and the rectified voltage *V_rect_* (**a**) in the P-SSHI mode, and (**b**) in the S-SSHI mode.

The first experiment is to verify the operation of the proposed circuit. The shaker is excited at 70 Hz. The open circuit voltage *V_oc_* is observed as around 5 V. Figure 13 shows the measured waveform of the PE voltage *V_BA_* and rectified voltage *V_rect_*. Figure 13a shows the proposed circuit operating in the P-SSHI mode. Here, we adjust the signal generator to double the *V_oc_* of the PE transducer for better observation under the P-SSHI mode. In the test, a resistor R_L_ of 220 kΩ is placed at the load to make the circuit operate in the P-SSHI mode. When the resonant circuit operates, the PE voltage suddenly rises from 4.25 V to 0.58 V, or drops from 4.25 V to 0.61 V. Then, the current charges the C_P_ with its voltage increase. As expected, the PE voltage is clamped by the rectified voltage *V_rect_* of 4 V. When the *V_oc_* is adjusted manually back to 5 V, the circuit enters the S-SSHI mode, and the waveform of the PE voltage is shown in Figure 13b. The PE voltage *V_BA_* instantly rises from 3.58 V to 0.25 V, or drops from 3.52 V to −2.9 V when the resonant circuits run. The PE transducer is in an open circuit most of the time due to high unreachable rectified voltage.

**Figure 14:**
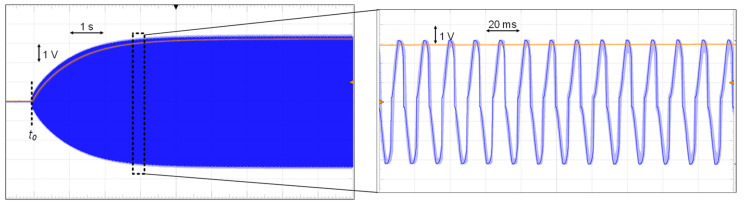
Cold start of the circuit.

Next, we tested the cold start of the proposed circuit. At *t_0_*, the excitation signal is applied to drive the shaker to excite the PE transducer. As shown in Figure 14, after *t_0_*, the PE voltage amplitude increases slowly from 0 V to 3 V, and so does the rectified voltage *V_rect_*. The zoom-in waveform on the right shows the detail. It verifies that the circuit has the capability of cold start. 

**Figure 15:**
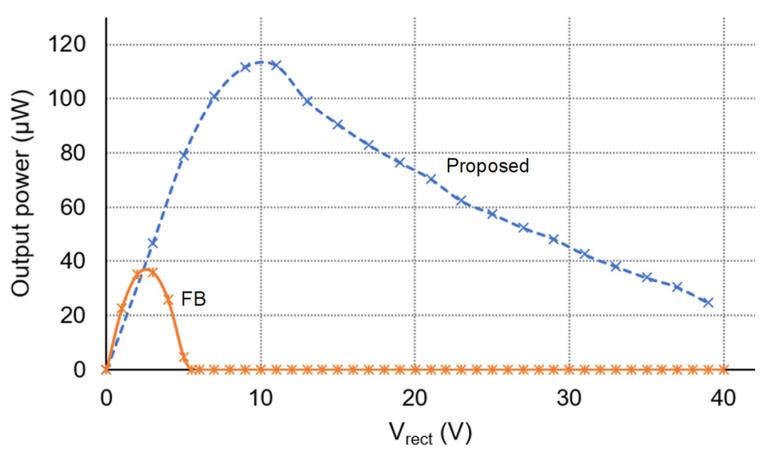
Power delivered to the load for the full-bridge (FB) circuit and the proposed circuit.

**Figure 16:**
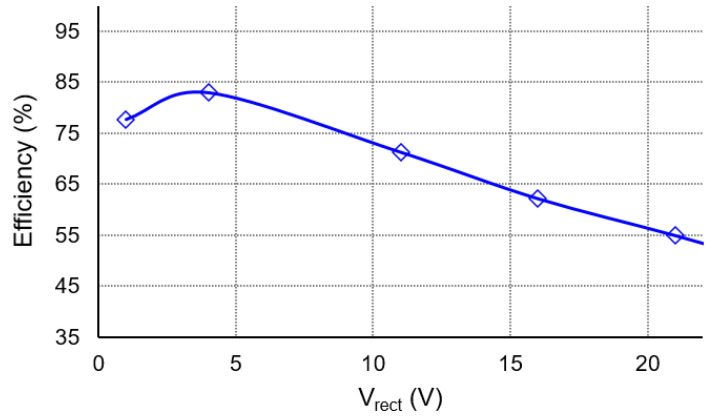
Power conversion efficiency of the circuit.

Figure 15 shows the power delivered to the load using two different circuits, the FB circuit and the proposed circuit, with the rectified voltage *V_rect_* ranging from 0 V–40 V. The open circuit voltage is observed as 5 V. The excitation frequency is set to 70 Hz. There is an optimal rectified voltage for the two circuits, which is 2.5 V for the FB rectifier and 11 V for the proposed circuit, in which the FB rectifier and proposed circuit can deliver the maximum power of 35 μW and 112 μW, respectively. Consider the VRI when *m* = 2 for the ratio of the half-maximum power to the maximum power obtained by the load. The proposed circuit has the wider operational rectified voltage range. The VRI of the proposed circuit is 4.4, which is 6.5 times higher than that of the FB rectifier. As the *V_rect_* increases, the output power decreases in the S-SSHI mode, and this may be caused by the losses increase in the circuit, which is evident from Figure 16. The power conversion efficiency is up to 83.2%. To understand the circuit’s loss breakdown, we simulated the circuit using the simple model parameters of *I_P_* = 70 μA, C_P_ = 22 nF and R_P_ = 800 kΩ. The majority loss of the proposed circuit is from passive diodes, about 15.3 μW on average. Due to the use of non-ideal switches of transistors Q_3_ and Q_4_, the second-most loss source is about 7 μW on average. Note that transistors of non-idealities would affect the circuit’s performance, thereby deviating from the theoretical analysis in Section 3.4. For example, the non-idealities may cause the output power of the proposed circuit in the S-SSHI mode to be lower than that of the SECE circuit. 

Table 3 summarizes the performance and characteristics of recent, state-of-the-art PE energy harvesting circuits. Consider the weakly coupled PE transducer. The figure of metric (FoM) in [13] compares the measured output power by the circuit against the maximum output power obtained by an ideal FB rectifier to estimate the proposed circuit’s power extraction capability.

(19) $$FoM=\frac{P_{out}}{f_{P}C_{P}V_{oc}^{2}}$$

Among the five designs with different power extraction schemes shown in Table 3, they all adopt nonlinear techniques. The proposed circuit and [15] have the highest FoM of 2.9. Thanks to the hybrid SSHI, the VRI of the proposed circuit is much higher than the other reported circuits. The proposed circuit is simple and has a minimum number of components—only eleven. The power conversion efficiency is up to 83.2%, which could be further improved by the adoption of active diodes.

## 6. Conclusions

This paper presents a rectification scheme that uses two resonant circuits to flip the PE transducer’s internal capacitor voltage. The proposed circuit integrates the hybrid SSHI circuit with a diode bridge rectifier, which operates either in the P-SSHI mode or in the S-SSHI mode according to the PE voltage. Only eleven components implement the circuit. Thanks to the rectification scheme, the circuit can efficiently harvest energy, even if the rectifier voltage is quite high. A PE cantilever mounted on the shaker was used to test the operation and performance of the circuit. The experiment results show that the VRI of the proposed circuit is 4.4 times higher than that of the FB rectifier, and the maximum output power is 2.9 times that of the ideal FB circuit. The power conversion efficiency is up to 83.2%. Future work will focus on implementing the proposed scheme using the CMOS process, which would reduce power dissipation using active diodes and lower-power controllers and avoid some adverse effects due to discrete devices of non-idealities.

## Figures and Tables

**Table 1:** Simulation parameters.

Components	Value
Piezoelectric transducer current *i_P_*	50 μA
Frequency *f_P_*	100 Hz
Capacitor C_P_	19 nF
External inductor L	1.5 mH

**Table 2:** Comparison of the VRI of the three different circuits.

VRI		
P-SSHI	S-SSHI	Proposed
$\frac{\sqrt{1-\frac{1}{m}}}{1-\eta_{F}}$	$\sqrt{1-\frac{1}{m}}$	$\frac{2}{1-\frac{m}{\sqrt{2}+1}}-\frac{1-\sqrt{1+\frac{2\left(1-\sqrt{2}\right)}{m}}}{3-2\sqrt{2}}$

**Table 3:** Comparison of recent PE energy harvesting circuits.

Publication	JSSC 2015 [3]	TPE 2015 [12]	TPE 2015 [15]	JSSC 2019 [16]	This work
Process Technology	0.35 μm CMOS	Discrete Components	Discrete Components	0.18 μm HV CMOS	Discrete Components
PE Transducer	Mide V21BL	Mide V22B	Custom	Custom MEMS	Custom
*C_P_* (nF)	11	18	28.42	1.94	22
*f_P_* (Hz)	200	225	24.9	219	70
*V_oc_* (V)	1–7	2.4	15	2.5	5
Number of Components	-	24 ***	19	-	11
Extraction Scheme	Dual-mode	SSHI	Triple Bias-flip	SE-SSHC	Hybrid SSHI
Self-powered	Yes	Yes	Yes	Yes	Yes
VRI *	<2.33 (V_oc_ = 3V) **	1.67 **	0.533 **	1.76 **	4.4
FoM	-	2.06	2.9 **	2.56	2.9
Power Conversion Efficiency	<80%	-	-	-	83.2%

* *m* = 2. ** Calculate from the paper. *** The count includes the number of logical units, such as NOR gates, AND gates, comparators, etc.

## Data Availability

No new data were created or analyzed in this study. Data sharing is not applicable to this paper.
